# Locked Mouths: Tooth Loss in a Women's Prison in Northeastern Brazil

**DOI:** 10.1155/2014/587469

**Published:** 2014-07-08

**Authors:** Iris Sant' Anna Araújo Rodrigues, Ingrid Thays de Melo Silveira, Magaly Suênya de Almeida Pinto, Alidianne Fabia Cabral Xavier, Thaliny Batista Sarmento de Oliveira, Saul Martins de Paiva, Ricardo Dias de Castro, Alessandro Leite Cavalcanti

**Affiliations:** ^1^Post Graduate Program in Public Health, State University of Paraiba, Avenida das Baraunas, S/N, Bodocongo, 58429-500 Campina Grande, PB, Brazil; ^2^Department of Dentistry, Faculty of Dentistry, State University of Paraiba, Avenida das Baraunas, S/N, Bodocongo, 58429-500 Campina Grande, PB, Brazil; ^3^Department of Pediatric Dentistry and Orthodontics, Faculty of Dentistry, Universidade Federal de Minas Gerais, Avenida Antonio Carlos 6627, Campus Pampulha, 31270-901 Belo Horizonte, MG, Brazil; ^4^Department of Dentistry, Faculty of Dentistry, Federal University of Paraiba, Cidade Universitaria, Castelo Branco, 58051-900 Joao Pessoa, PB, Brazil

## Abstract

*Background*. Prisoners, in general, exhibit unsatisfactory oral conditions, especially with regard to the large number of missing teeth and with untreated caries. The aim of this study was to assess tooth loss, use of and need for prosthetic rehabilitation, and use of dental services among inmates. A cross-sectional study involving 65 inmates was developed at the Regional Women's Prison of Campina Grande, Brazil. Data regarding sociodemographic and sentencing profile, use of dental services, dental morbidity, and self-perceived oral health impacts were investigated. Chi-square, Pearson, and Kruskal-Wallis (*P* < 0.05) statistical tests were used. The mean tooth loss was 11.3 teeth. Significant association between tooth loss and oral health satisfaction (*P* = 0.049), self-perceived need for dental prosthesis (*P* < 0.001), uncomfortable teeth brushing (*P* = 0.005), difficult speaking (*P* = 0.002), and difficulty in performing routine tasks (*P* = 0.025) was observed. It was observed that 29.2% of inmates were using some type of prosthesis, all deemed unsuitable for use, and 78.5% of inmates needed prosthetic rehabilitation. The oral health condition of the population studied was found to be poor, and prisoners showed significant tooth loss and need for dentures, with the aggravation of having tooth extraction as the major reason for seeking dental care.

## 1. Introduction

Entering spaces delimited by walls, bars, doors, and finally people is not an easy task, which perhaps justifies the scarcity of studies addressing the oral health of the prison population in Brazil. However, given the increasingly pronounced inclusion of women in the prison environment, there is an increasing need for attention towards the oral health of this group since, neglecting the details of law, the penalties for drug trafficking and crimes against property, key drivers of female prison, vary from 1 to 15 years in prison, which is sufficient to rehabilitate or neglect the oral health of those resigned to the penalty of freedom deprivation [1].

According to the latest data provided by the National Penitentiary Department, Brazil has approximately 31,500 women under the custody of the prison system, representing approximately 6.2% of the total Brazilian prison population [2]. Although the scarcity of studies limits the knowledge about the oral health conditions of prisoners, international findings [3–6] have shown that prisoners, in general, exhibit unsatisfactory oral conditions, especially with regard to the large number of missing teeth and with untreated caries, justified by social exclusion and unemployment experienced prior to prison [7], associated with irregular use of dental services and use of drugs [8].

Studies aimed at caries experience in the general population have shown that females with low education and low income have greater propensity to dental loss [9]. This scenario is consistent with the profile shown by Brazilian prisoners since the female prison population in the five regions of the country is predominantly composed of women with low educational level, poor, unemployed or underemployed before prison, under 30 years of age, and whose arrest was motivated by illicit trafficking of narcotics followed by crimes against property [1].

Tooth loss, considered a mark of social and health inequities, results from the crippling dental practice, which due to the lower cost and greater “efficiency” remains among people of low socioeconomic level [9] and consequently those collected in prison environments. Considered as a public health problem [10], tooth loss can cause many problems, ranging from functional disorders such as difficulty in chewing and speaking to psychosocial problems, among which socialization difficulty stands out [11].

Considering that data on the subject are still scarce in the Brazilian literature, this study aimed to evaluate tooth loss, use of and need for prosthetic rehabilitation, and use of dental services among inmates of the Regional Women's Prison of Campina Grande, Brazil.

## 2. Materials and Methods

### 2.1. Study Population

This study was conducted in the city of Campina Grande, inner state of Paraiba, northeastern Brazil. The female prison population of the state is approximately 450 inmates, distributed into three prisons, designed for the imprisonment of women, located in the state capital, Joao Pessoa, and in the municipalities of Campina Grande and Patos.

This cross-sectional study was conducted at the Regional Women's Prison of Campina Grande. All women who were incarcerated in June 2012 were selected, totaling 67 women. The sample, however, was composed of 65 women, since two of them refused to participate.

Mediated by a prisoner who for good behavior renders services in the prison, the other inmates were invited to participate in the study. Thus, at every shift, five inmates were escorted by prison officers to the room dedicated to the survey. The data collection instrument was adapted for the study population based on the 2010 National Oral Health Survey (SB Brazil) using criteria recommended by WHO to asses tooth decay experience [12].

### 2.2. Training and Calibration Process

Intraoral clinical examination was performed by a single examiner, properly calibrated (Kappa 0.92), aided by an annotator properly trained for this function, both wearing apron to meet general biosecurity standards. For the examination of tooth surfaces, disposable wooden spatulas, gauzes, mouth mirrors, and properly sterilized millimeter probes (Community Periodontal Index CPI) were used.

### 2.3. Nonclinical Data Collection and Dental Examinations

Data were collected by interview and clinical examination. In the first part of the instrument, data regarding sociodemographic and sentencing profile of prisoners were collected, including variables age, educational level, labor activity prior to arrest, and jail time, as well as use of dental services, oral morbidity, and self-perceived and oral health impacts in the past six months. In the second part, aspects related to dental clinical examination were addressed, in which tooth decay experience and use of and need for prosthesis were assessed.

Although tooth loss assessment is very broad, this review sought to evaluate dental loss compared to DMFT and its other components, decayed and filled teeth in the four age groups, obtained by age quartile of female prisoners. For the bivariate analysis, tooth loss was dichotomized by the medians (≤8, >8), which were considered dependent variables: toothache for the last six months, oral health satisfaction, self-perceived need for dental treatment, and dental prosthesis and oral health impact in the last six months, whose variables are uncomfortable teeth brushing, difficulty and embarrassment while talking and smiling, difficulty performing routine activities, and difficulty in sleeping.

Use of dental prosthesis was considered as any prosthetic piece installed for prosthetic rehabilitation of existing spaces. The need for prosthesis was based on the presence of prosthetic spaces without rehabilitation or possibly left after performing teeth extractions and in the case of existing prostheses unsuitable for use. Prostheses presenting no aesthetics, retention, stability, and adequate fixation were deemed unsuitable for use [12].

### 2.4. Data Analysis

Data were organized and analyzed with the aid of SPSS (Statistical Package for Social Sciences), version 17, using descriptive statistics to obtain absolute and percentage distributions and analytical statistics for statistical calculations. To evaluate association between categorical variables, significance level of 5% (*P* < 0.05) and CI 95% were adopted. Pearson chi-square statistical tests or Fisher's exact and Kruskal-Wallis tests with comparisons were used.

### 2.5. Ethical Aspects

This study followed ethical guidelines recommended by the Brazilian legislation and was approved by the Human Research Ethics Committee of the State University of Paraiba. All participants/guardians signed the informed consent form.

## 3. Results

Analyzing the sociodemographic and sentencing profile, it was observed that the average age of inmates was 32.2 (±11.56) years, with a greater number of inmates within the age group of 19–24 years. As for schooling, most inmates (73.8%) failed to complete basic education. When asked about the performance of any labor activity prior to arrest, more than half (56.9%) answered affirmatively. The mean imprisonment period was 13.51 (±13.65) months, and most of inmates (52.4%) had been prisoners for a period equal to or less than 8 months (Table 1).

Regarding the use of dental services, only one prisoner reported that she had never been to an oral surgeon in her life. Of those who had, 71.9% had the last dental consultation less than one year ago, with half of the consultations being held in the prison service and with tooth extraction being the reason for the last visit for 40.6% of inmates (Table 2).

Tooth loss analysis revealed that only four inmates (6.1%) had no missing teeth. The majority of the sample (55.4%) had between one and ten missing teeth, 15.4% had 11–20 teeth lost, 12.4% had between 21 and 31 missing teeth, and seven prisoners (10.7%) were totally edentulous. The mean tooth loss was 11.26 (±10.38) teeth.

Table 3 shows that tooth loss increased according to age, with the highest averages in the age groups from 31 to 38 years (13.00 ± 8.94) and greater than 39 years (22.63 ± 10.18), respectively. After tooth caries, tooth loss was the component that contributed most to the DMFT values in the age groups from 19 to 24 years and from 25 to 30 years. In the age group from 31 to 38 years and greater than 39 years, tooth loss was the component that contributed most to the DMFT values, followed by decayed and filled teeth, respectively. In the total group, tooth loss showed the highest average (11.26 ± 10.38), followed by decayed components (7.83 ± 5.01) and filled teeth (1.28 ± 2.58).

The results in Table 4 showed significant association (*P* < 0.005) between tooth loss and oral health satisfaction, self-perceived need for dental prosthesis, pain when drinking hot and/or cold liquids, uncomfortable teeth brushing, difficulty in speaking, and difficulty in performing routine tasks.

Regarding the use of dental prosthesis, it was observed that 29.2% of inmates were using some type of prosthesis, all considered unsuitable for use. Of prostheses in use, 52.6% were partially removable, installed in the upper arch. Regarding the need for prostheses, 78.5% of inmates needed prosthetic rehabilitation. Many of them needed partial rehabilitation of upper and lower teeth (62.7%), followed by complete rehabilitation of upper and lower teeth (13.7%) (Table 5).

## 4. Discussion

Until the moment, this is the first study in Brazil that described the tooth loss, use of and need for prosthetic rehabilitation, and use of dental services among female inmates. Due to the growth of the female prison population found in virtually all states of Brazil [1] and the poor oral health of prisoners [13], there is need to understand what are the main dental needs of this group to serve as base to the reflection on the topic and to encourage the development of new studies aiming to contribute to the formulation and improvement of dental care policies in the prison environment, thus collaborating with a higher quality of life and health and consequently with the social reintegration of women coming from the penitentiary system.

It is interesting to observe how the relationship between tooth loss and prison environment ends up, in most cases, by referring to the same group of individuals, that is, those from the social groups marked by lack of opportunity and social protection. In this perspective and analyzing the profile of prisoners under study, it is observed that as in all states of Brazil [1], young women with low educational level, who prior to prison were unemployed or badly paid, are those who agglomerate in women's prisons.

Individuals with lower income and educational level show a trend of irregular use of dental services, which consequently express an unfavorable reality of their oral condition resulting from the difficult access to information and to effectively resolve dental services [8]. In this sense, it was observed that low education interferes with many aspects since, in addition to preventing activities that provide higher incomes and consequently the access to private services, this predisposes individuals to lower health conditions, leading to attitudes and behaviors that result in poorer oral health.

Evaluating the average jail time, it was concluded that possibly tooth loss cases in this group of prisoners are a result of lack of oral health care prior to arrest, since such a large amount of tooth loss in a short jail time period is not justified without previous neglect. However, there is a limit on the certification of this understanding: first, because it is a cross-sectional study; second, due to the absence of dental records of treatments performed during the jail time, which prevents the comparison between the oral health condition upon entry into prison and the period of data collection.

Regarding the aspect use of dental services, it was observed that most of the inmates visited the dentist in the last year; however, a considerable number of consultations were to perform tooth extraction. However, although extracted dental elements showed no pain symptoms on the day of the last visit, many of the inmates reported history of pain at an earlier time, which motivated extractions precisely to avoid recurrence of pain, since emergency dental visits in this prison unit are not always readily resolved, notably due to the lack of dental office installed in the penitentiary.

According to the National Health Plan in the Prison System in Brazil, prison units with less than 100 inmates should refer their prisoners to prisons provided with health units in their facilities [1]. It is the case of the Women's Prison of Campina Grande, whose inmates receive dental care in male penitentiaries located in the same prison complex. Thus, some limitations can be found in the provision of dental services to these women, because this service requires correctional officers to perform escort and transportation, which availability sometimes is limited.

In this regard, it is understood that the limited access to preventive and treatment measures resulting from the lack of dental care planning ends up by resulting in mutilating treatments because it is the only technically possible procedure regarding the disease severity, relief of pain, and lack of access to other types of treatment [14]. However, one should consider that the decision to rehabilitate or not a tooth element is also related to other aspects such as the self-perceived importance of maintaining tooth elements, which is closely linked to cultural factors and access to information, as well as to past experiences with ineffective rehabilitation treatments [11]. Therefore, health promotion activities inside prisons should be encouraged, seeking to clarify and encourage the preservation of teeth as well as the effective access and referral to specialized centers for cases previously requiring more complex assistance.

Evaluating tooth loss as a result of a treatment option, that is, tooth extraction, it appears that the average number of missing teeth among prisoners of Campina Grande was well above the average number of filled teeth in all age groups. Study with 500 prisoners from a prison in the United States found higher average number of missing teeth in relation to filled teeth only in the age group over 30 years [15], although this difference does not seem to be higher than that found in this study. In this sense, there is questioning about the actual effectiveness of measures proposed by the National Health Plan in the Prison System, which is to provide dental service based on primary care and focused on measures to promote and protect the health of prisoners.

Also in this respect, it is noteworthy that tooth loss is a nationwide concern because although there has been a drop in the number of missing teeth and an increase in the number of filled teeth, tooth loss still remains the most responsible for the high DMFT values in the age groups from 35 to 44 and from 65 to 74 years, as can be seen in the results of nationwide oral health survey conducted in 2010 [16]. Although an immediate comparison of findings obtained in this study with data from the Brazilian population is not possible due to the incompatibility of age groups, it should be noted that this reality extends the inmates of the Women's Prison of Campina Grande, since, among the DMFT components, tooth loss showed the highest average in the age group over 30 years.

Regarding tooth loss, it was observed that the number of present teeth directly affects the quality of life of individuals, which can be observed among women in this study, in which those with more than eight missing teeth were less satisfied with their oral health and showed more difficulty in speaking, uncomfortable teeth brushing, and difficulty in performing routine activities. Furthermore, it is noteworthy that the problems arising from the lack of teeth can include not only functional limitation but also the aesthetic appearance, showing a poorer socioeconomic condition, which may have an important influence on employment, social and cultural relationships, and the self-esteem of people [17].

However, this reality needs to be changed, since according to the contemporary definitions of health, oral health presents other perspectives, which are understood as a comfortable and functional dentition, with an appearance that allows individuals to fulfill their social function and daily activities without physical, psychological, or social disorders. Therefore, it should be understood that the oral health rehabilitation of individuals deprived of freedom can play a key role in social reintegration.

Regarding tooth loss rehabilitation, it was found that although a large number of prisoners perceived and needed prosthetic rehabilitation, only a small portion of this group made use of prostheses, all unsuitable for use. However, this is a very common reality among economically disadvantaged individuals that, given the impossibility of restoring tooth loss by the use of prostheses mainly due to lack of financial resources and access to dental services, ends up by not using them or by using them for a time that extends far beyond the recommended period for due replacement [17].

Although the implementation of the Oral Health Policy in Brazil has considerably improved the access to dental care services, there is still a large portion of the population marked by mutilating procedures and without access to prosthetic rehabilitation. However, although public health services still remain to be desired, it is necessary to emphasize that, for the prison population, dental care service should be guaranteed and focused on truly effective preventive and rehabilitative measures, due to both the existence of a Penitentiary Health Insurance, which includes dental care, and the limiting access to health services outside prison, as individuals in custody are resigned to the power of the state and prison authorities.

## 5. Conclusion

The oral health status of the population studied was found to be poor and inmates showed considerable amounts of tooth loss and need for dentures, in which tooth extraction was the most important reason for seeking dental care. Given this context, there is need for better organization of the prison system in order to improve dental services and allow greater resolution and efficiency of the dental assistance aimed at this population. Therefore, there is need for the implementation of actions intended to inform and promote oral health and prevent further tooth loss and prosthetic rehabilitation in order to restore the oral health of these women and thus to contribute to their social reintegration.

## Figures and Tables

**Table 1:** Distribution of inmates according to sociodemographic and sentencing profile.

Variables	Frequency		Mean ± SD
	*n*	%	
(1) *Sociodemographic *			
Age (years)			32.2 (11.56)
Quartile 1 (19–24 )	18	27.7	
Quartile 2 (25–30)	16	24.6	
Quartile 3 (31–38)	15	23.1	
Quartile 4 (39 or older)	16	24.6	
Total	**65**	**100**	
Educational level			
Illiterate	2	3.1	
Complete basic education	48	73.8	
Incomplete basic education	6	9.2	
Complete high school	6	9.2	
Incomplete high school	2	3.1	
Incomplete higher education	1	1.5	
Complete higher education	0	0.0	
Total	**65**	**100**	
Labor prior to arrest			
Yes	37	56.9	
No	28	43.1	
Total	**65**	**100**	
(2) *Sentencing profile *			
Jail time (months)			13.51 (13.65)
1–5	17	26.2	
6–8	17	26.2	
9–17	17	26.1	
18–60	14	21.2	
Total	**65**	**100.0**	

**Table 2:** Distribution of inmates according to the use of dental services.

Variable	Frequency	
	*n*	%
Have been to the dentist at least once in life?		
Yes	63	96.9
No	1	1.5
Does not know/Did not answer	1	1.5
Total	**65**	**100.0**
When was the last visit to the dentist?		
Less than one year	46	71.9
Between 1 and 2 years	3	4.7
Three or more	12	18.8
Does not know/Did not answer	3	4.7
Total	**64**	**100.0**
Where was your last visit?		
Prison	32	50
External Public Service	10	15.6
Private Service	20	31.3
Does not know/Did not answer	2	3.1
Total	**64**	**100.0**
Reason for last visit?		
Prevention	1	1.6
Pain	4	6.3
Extraction	26	40.6
Restorative treatment	22	34.4
Others	9	14.1
Does not know/Did not answer	2	3.1
Total	**64**	**100.0**

**Table 3:** Tooth loss assessment according to age group, DMFT index, and decayed and filled components.

	Age groups				Total	*P* value
	19–24	25–30	31–38	>39		
	Mean ± SD median	Mean ± SD median	Mean ± SD median	Mean ± SD median	Mean ± SD median	
Decayed	9.72 ± 4.14^(A)^	7.87 ± 4.92^(AB)^	9.07 ± 5.19^(A)^	4.50 ± 4.53^(B)^	7.83 ± 5.01	0.020
	(9.50)	(7.50)	(8.00)	(3.50)	(8.00)	
Lost	3.28 ± 3.25^(A)^	7.25 ± 5.89^(B)^	13.00 ± 8.94^(C)^	22.63 ± 10.18^(D)^	11.26 ± 10.38	<0.001
	(2.00)	(6.00)	(10.00)	(24.00)	(8.00)	
Filled	2.22 ± 3.19^(A)^	2.00 ± 3.52^(A)^	0.60 ± 0.99^(AB)^	0.13 ± 0.34^(B)^	1.28 ± 2.58	0.038
	(0.00)	(1.00)	(0.00)	(0.00)	(0.00)	
DMFT	15.22 ± 5.61^(A)^	17.13 ± 8.06^(A)^	22.67 ± 5.63^(B)^	27.25 ± 5.94^(C)^	20.37 ± 7.87	<0.001
	(14.50)	(20.00)	(23.00)	(29.00)	(21.00)	

^+^Distinct letters in brackets demonstrate significant differences between corresponding age groups.

**Table 4:** Relationship between tooth loss, tooth pain, oral health satisfaction, self-perception, and impact.

Variable	Tooth loss						*P* value	RP (CI 95%)
	9 to 32		Up to 8 (median)		Total			
	*n*	%	*n*	%	*n*	%		
Pain in the last 6 months								
Yes	12	50.0	22	64.7	34	58.6	0.263	1.00
No	12	50.0	12	35.3	24	41.4		1.29 (0.81 to 2.07)
Total	**24 **	**100.0**	**34**	**100.0**	**58**	**100.0**		
Oral health satisfaction								
Satisfied	5	16.1	14	41.2	19	29.2	0.049	∗
Unsatisfied	24	77.4	16	47.0	40	61.5		∗
Nor satisfied/or satisfied	2	6.5	4	11.8	6	9.3		∗
Total	**31**	**100**	**34**	**100**	**65**	**100**		
Self-perceived need for dental treatment								
Yes	23	74.2	31	91.2	54	83.1	0.068	1.00
No	8	25.8	3	8.8	11	16.9		1.23 (0.97 to 1.55)
Total	**31**	**100**	**34**	**100**	**65**	**100**		
Self-perceived need for use of prosthesis								
Yes	24	77.4	5	14.7	29	44.6	<0.001	5.26 (2.29 to 12.09)
No	7	22.6	29	85.3	36	55.4		1.00
Total	**31**	**100**	**34**	**100**	**65**	**100**		
Oral health impacts								
Difficulty in eating								
Yes	11	35.5	11	32.4	22	33.8	0.790	1.10 (0.56 to 2.16)
No	20	64.5	23	67.6	43	66.2		1.00
Total	**31**	**100**	**34**	**100**	**65**	**100**		
Uncomfortable tooth brushing∗∗								
Yes	2	5.9	9	37.5	11	19.0	0.005	∗
No	32	94.1	15	62.5	47	81.0		
Total	**34**	**100**	**24**	**100**	**58**	**100**		
Difficulty in speaking								
Yes	8	25.8	—	—	8	12.3	0.002	∗
No	23	74.2	34	100	57	87.7		
Total	**31**	**100**	**34**	**100**	**65**	**100**		
Embarrassment while talking								
Yes	6	19.4	6	17.6	12	18.5	0.859	1.10 (0.39 to 3.05)
No	25	80.6	28	82.4	53	81.5		1.00
Total	**31**	**100**	**34**	**100**	**65**	**100**		
Embarrassment while smiling								
Yes	8	25.8	7	20.6	15	23.1	0.618	1.25 (0.51 to 3.05)
No	23	74.2	27	79.4	50	76.9		1.00
Total	**31**	**100**	**34**	**100**	**65**	**100**		
Difficulty in performing routine tasks∗∗								
Yes	—	—	4	16.7	4	6.9	0.025	∗
No	35	100	19	83.3	54	93.1		
Total	**35**	**100.0**	**23**	**100.0**	**58**	**100.0**		

*Unable to determine due to the occurrence of zero and very low frequencies. **Not applied to edentulous subjects.

**Table 5:** Distribution of prisoners according to the use of and need for dentures.

Variable	Frequency	
	*n*	%
Ever made use of prosthesis?		
Yes	19	29.2
No	46	70.8
Total	**65**	**100.0**
Prosthesis in use is appropriate?		
Yes	0	0
No	19	100.0
Total	**19**	**100.0**
What kind of prosthesis is in use?		
Unitary prosthesis	1	5.3
Upper and lower TP	3	15.8
Upper TP	3	15.8
Upper and lower RPP	1	5.3
Upper RPP	10	52.6
Lower RPP	1	5.3
Total	**19**	**100.0**
Need for dentures?		
Yes	51	78.5
No	14	21.5
Total	**65**	**100.0**
Upper and lower TP	7	13.7
Upper and lower RPP	32	62.7
Upper RPP	4	7.8
Lower RPP	5	9.8
Upper TP and lower RPP	3	5.9
Total	**51**	**100.0**

TP: total prosthesis; RPP: removable partial prosthesis.
